# Specific binding of eukaryotic ORC to DNA replication origins depends on highly conserved basic residues

**DOI:** 10.1038/srep14929

**Published:** 2015-10-12

**Authors:** Hironori Kawakami, Eiji Ohashi, Shota Kanamoto, Toshiki Tsurimoto, Tsutomu Katayama

**Affiliations:** 1 Department of Molecular Biology, Graduate School of Pharmaceutical Sciences, Kyushu University, 3-1-1 Maidashi, Higashi-ku, Fukuoka 812-8582, Japan; 2 Department of Biology, School of Sciences, Kyushu University, 6-10-1 Hakozaki, Higashi-ku, Fukuoka 812-8581, Japan

## Abstract

In eukaryotes, the origin recognition complex (ORC) heterohexamer preferentially binds replication origins to trigger initiation of DNA replication. Crystallographic studies using eubacterial and archaeal ORC orthologs suggested that eukaryotic ORC may bind to origin DNA via putative winged-helix DNA-binding domains and AAA+ ATPase domains. However, the mechanisms how eukaryotic ORC recognizes origin DNA remain elusive. Here, we show in budding yeast that Lys-362 and Arg-367 residues of the largest subunit (Orc1), both outside the aforementioned domains, are crucial for specific binding of ORC to origin DNA. These basic residues, which reside in a putative disordered domain, were dispensable for interaction with ATP and non-specific DNA sequences, suggesting a specific role in recognition. Consistent with this, both residues were required for origin binding of Orc1 *in vivo.* A truncated Orc1 polypeptide containing these residues solely recognizes ARS sequence with low affinity and Arg-367 residue stimulates sequence specific binding mode of the polypeptide. Lys-362 and Arg-367 residues of Orc1 are highly conserved among eukaryotic ORCs, but not in eubacterial and archaeal orthologs, suggesting a eukaryote-specific mechanism underlying recognition of replication origins by ORC.

Formation of higher-order nucleoprotein complexes at chromosomal replication origin(s) is a prerequisite for once-per-cell-cycle DNA replication in all three domains of life^1^,^2^,^3^,^4^. In eukaryotes, the earliest step of DNA replication is ATP-dependent association of the origin recognition complex (ORC), which consists of Orc1/2/3/4/5/6, with chromosomal replication origins. In yeast, these replication origins are called autonomous replicating sequences (ARSs). In G1 phase, ORC binding to replication origins triggers recruitment of Cdc6, Cdt1, and the MCM2–7 heterohexamer onto double-stranded (ds) DNA to form the ORC–Cdc6–Cdt1–MCM2–7 (OCCM) complex^5^. This step is followed by ordered dissociation and association processes, which allows formation of the ORC–Cdc6–MCM2–7 (OCM) intermediate and recruitment of another MCM2–7 hexamer to yield a salt-stable, head-to-head MCM2–7 double hexamer. The resultant double hexamer is the substrate for formation of the Cdc45–MCM2–7–GINS (CMG) complex at the G1–S transition. The CMG complex, which is an active helicase *in vitro*, is thought to serve in S phase as the replicative helicase component of the replisome for daughter-strand synthesis.

Basic structures of all of the ORC subunits except for Orc6 are suggested to be highly conserved among eukaryotes. Orc1/2/3/4/5 contain a motif related to the AAA+ (ATPase associated with various cellular activities) family in the middle region of each primary sequence, as well as one or two putative DNA-binding winged-helix (WH) motifs in the C-terminal region. A typical AAA+ fold is a globular structure with 5 parallel β-strands that make up a β-sheet, which is encircled by helical structures. A typical WH fold is a compact α/β structure bearing twisted antiparallel β strands and the two characteristic loops (a.k.a. ‘wings’) at the C-terminus of the fold^6^. Orc1, the largest subunit of ORC, has an extension at the N-terminus not found in other ORC subunits. The N-terminal half of the extension contains a bromo-adjacent homology (BAH) domain that has affinity for transcription-related proteins^7^. The BAH domain in *Saccharomyces cerevisiae* (Sc) Orc1 is a globular structure in the crystal, consisting of a conserved BAH core rich in β-strands and a non-conserved helical sub-domain^8^. The BAH domain is dispensable for cell viability in *S. cerevisiae*, but stabilizes the association between ORC and a subset of ARSs *in vivo*^9^. Mechanism of this stabilization process might not be simple, as another group shows that ScORC-ARS binding is stabilized by nucleosome *in vitro* and that the BAH domain is dispensable for this process^10^. So far, more than 400 ARSs throughout the genome have been experimentally confirmed^11^. Comprehensive mutagenesis of ARS plasmids has revealed two major functional elements of these sequences: the essential A element containing the ARS consensus sequence (ACS) and the stimulatory B elements^12^.

ScORC binds specifically to ARSs *in vitro* in a competitor DNA-dependent manner^13^,^14^. By contrast, in *Schizosaccharomyces pombe* (Sp), *Drosophila* (Dm), and human (Hs), ORC-origin DNA binding is less sequence-specific, with a preference for AT-rich or negatively supercoiled DNA^15^,^16^,^17^,^18^. In light of this difference, ScORC represents a unique model for analyzing the crucial mechanisms that support molecular recognition of ARSs.

ORC orthologs in eubacteria and archaea have properties similar to those of eukaryotic ORC. In *Escherichia coli*, DnaA binds to the unique chromosomal replication origin *oriC* to recruit DnaB helicase^4^. Like Orc1, DnaA has a protein–protein interaction domain at the N-terminus, an AAA+ domain in the middle region, and a helix-turn-helix (HTH) DNA-binding domain (a simplified WH) at the C-terminus. An unstructured linker connects the N-terminal and AAA+ domains. The HTH domain directly binds to DNA^19^. In archaea, *Sulfolobus solfataricus* has three ORC orthologs, two of which form a heterodimer on one of three replication origins. By contrast, *Aeropyrum pernix* has only one ORC ortholog. In crystal structures of both archaeal proteins, the WH and AAA+ domains contact origin DNA^20^,^21^; the WH domain determines affinity for origin DNA, whereas the AAA+ domain contributes to origin specificity^22^.

In contrast to the situation in eubacteria and archaea, the common mechanisms by which eukaryotic ORC recognizes replication origins remain unknown. Requirements of AAA+ and WH domains for eukaryotic ORC binding to ARS have not been reported. Mechanism in BAH domain-dependent stimulation of ScORC-ARS binding *in vivo* remains elusive. Indeed, the mechanisms experimentally verified to date have emphasized diversity rather than similarity among species. In *Drosophila*, the least conserved subunit of ORC, Orc6, is essential for DNA binding, whereas ScORC lacking Orc6 can bind to ARS and recruit Cdc6^23^,^24^,^25^. Hs Orc6 has little affinity for Orc1/2/3/4/5, yielding a substoichiometric hexamer *in vitro*^26^. In *S. pombe*, the predominant DNA-binding domain of ORC is in the N-terminal extension in Orc4, which is not conserved in other eukaryotes^17^,^18^,^27^. Recently, we performed single-particle analysis of the Cdc6–ORC–ARS complex in *S. cerevisiae*, on the basis of which we proposed that ARS tracks along the interior surface of the complex; this model is consistent with previous studies using different approaches, such as *in vitro* subunit mapping by crosslinking and observations of DNA bending by ScORC^24^,^28^,^29^. Given that eubacterial DnaA and archaeal ORC bind origin DNA(s) via their HTH and WH domains, respectively, with a contribution from the AAA+ domain in the case of archaeal ORC, some researchers have inferred that eukaryotic ORC may also bind origin DNA via WH and AAA+ domains^3^,^7^,^26^. So far, none of these hypotheses have been experimentally validated using eukaryotic mutant proteins.

In this study, we predicted a domain of ScORC involved in ARS binding, based on the structural model described above. Mutational analysis of this domain revealed that two residues play crucial roles in the specific interaction between the ARS and ORC hexamer. Contrary to the hypotheses described above, the identified residues are located in a basic residue-rich region outside the WH and AAA+ domains. Because these residues are highly conserved among eukaryotes, we propose a general mechanism in which eukaryotic ORC recognizes replication origins via the basic patch in Orc1, which we refer to as the eukaryotic origin sensor (EOS).

## Results

### Prediction of ORC residues that interact with the essential element of *ARS1*

**Figure 1:**
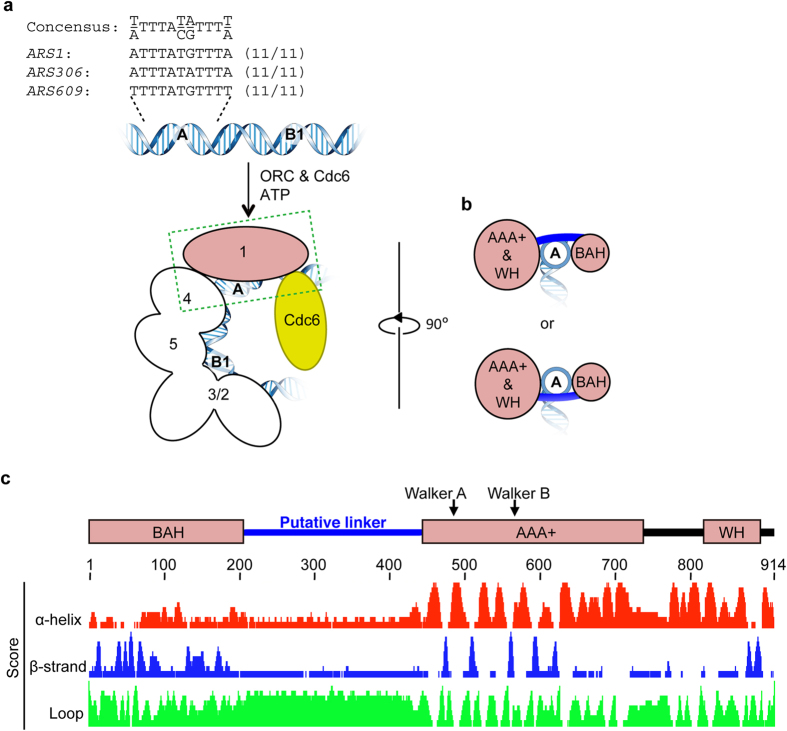
Structural models of ScOrc1. (**a**) Cartoons summarizing the ORC binding regions in typical ARSs and the structural model of the ORC-Cdc6-*ARS1* complex^5^,^28^. ACSs in *ARS1, ARS306*, and *ARS609* are shown on top. Orc1 and Orc2/3/4/5 are shown in pink and white, respectively. Possible locations of the A and B1 elements are indicated. For clarity, Orc6 and the WH domains of Orc2/3/4/5 are omitted. (**b**) Side view of Orc1 complexed with DNA (as indicated by a dotted rectangle in panel (**a**) rotated 90° around the vertical axis from panel (**a**). Possible loci of a linker region that connects BAH and AAA+ domains are indicated as blue lines. (**c**) Domain structure and prediction of secondary structure of ScOrc1. Representative domains and motifs are indicated along with the predicted score of each residue. Walker A and B indicate the ATP-binding motifs. See text for BAH, AAA+ and WH.

To identify the subunits, domains, and residues of ORC essential for ARS binding, we first examined the structural model of the ORC–Cdc6–*ARS1* DNA complex we reported previously^5^,^28^. The AAA+ and WH domains of Orc1 are located on the same surface of the elongated, crescent-like, two-lobed shape, whereas the BAH domain is located on the other side of the structure (Fig. 1a,b). *ARS1* is located in the center of ORC, tracking along the interior surface; the A element of *ARS1* is close to the Orc1 subunit, consistent with the results of an *in vitro* mapping experiment in which purified ORC and *ARS1* were incubated in the presence of a crosslinker^24^. We noticed that the putative linker domain of Orc1, not mentioned in the original reports, should be located between the BAH and AAA+ domains, and that the A element of *ARS1* would thus be near the linker domain as well as the AAA+ and WH domains (Fig. 1b,c).

**Figure 2:**
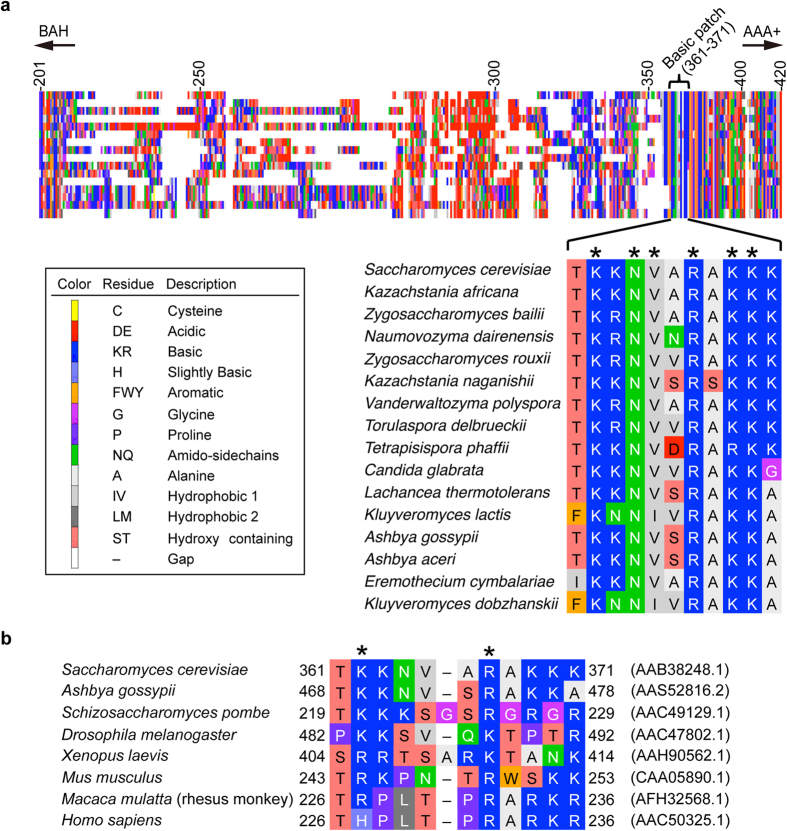
*In silico* analyses of Orc1. (**a**) Multiple alignment of a BLAST search with ScOrc1^201–420^ is shown on top using a colored rectangular pixel for each residue. A magnified alignment of the basic patch is shown below. The residues assigned to each color are indicated by the legend at left *, conserved residues. Full details of the alignment are shown in Supplementary Data S1. (**b**) Multiple alignment of the basic patch of eukaryotic Orc1 homologs, including representative model species. NCBI accession numbers are indicated on the right. Residues are colored as in panel (**a**). Histidine, though less basic than lysine or arginine, can play a crucial role for DNA binding at physiological pH^19^. *, Lys-362 and Arg-367 residues in ScOrc1 and equivalents in homologs.

If this hypothesis is true, the linker domain may contain residues that could contact *ARS1*. To test this idea, we performed an *in silico* screen to identify conserved residues in the linker domain. BLAST search and multiple alignment analysis unveiled an 11 amino acid “patch” (ScOrc1^361–371^) conserved in fungi that is rich in basic residues (i.e., the EOS; Fig. 2a and Supplementary Data S1). A similar basic patch is also present in a wide range of eukaryotic cells including fungi, metazoans, and human, but not in representative eubacterial and archaeal orthologs (Fig. 2b and Supplementary Fig. S1). Because basic residues are positively charged at physiological pH, and DNA is negatively charged, the EOS is likely to contain DNA-binding residues.

### Orc1 residues Lys-362 and Arg-367 have a crucial role for ARS-binding *in vivo*

**Figure 3:**
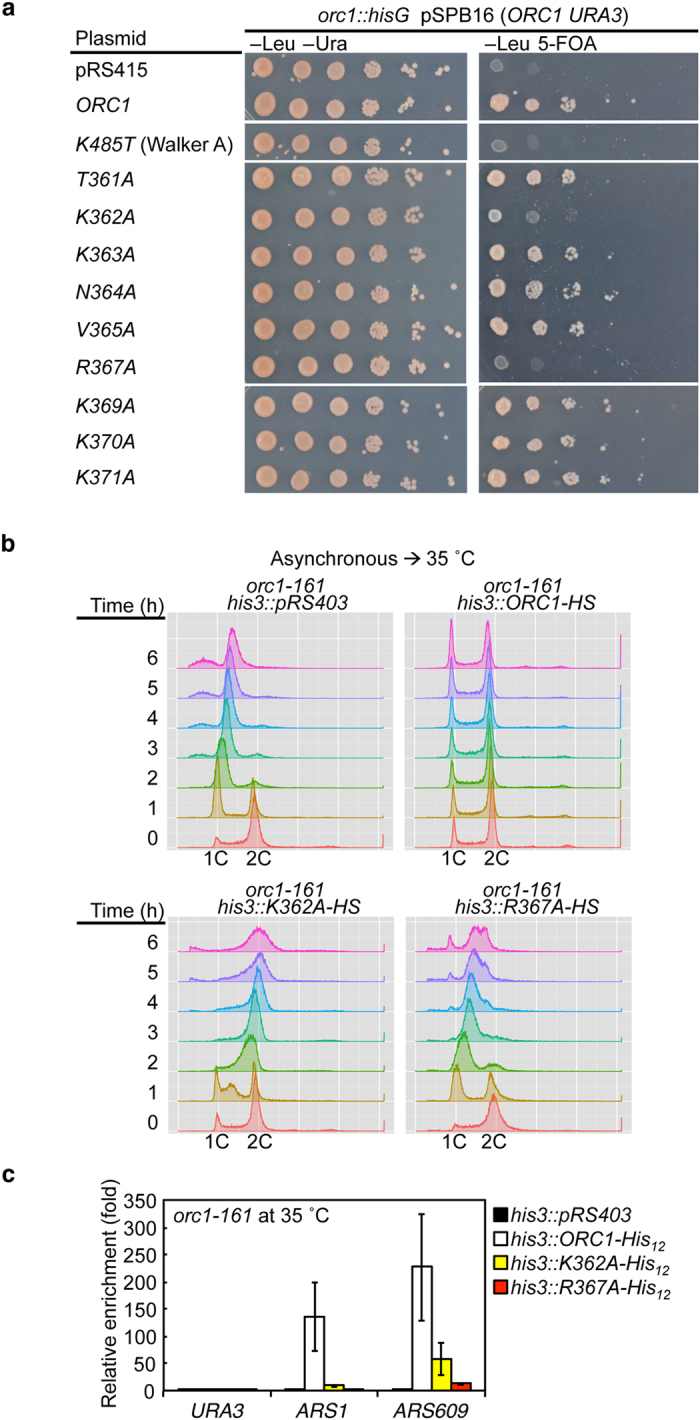
*In vivo* activities of *orc1* mutants. (**a**) Plasmid-shuffle assay. The tester strain YB838 (*orc1::hisG* pSPB16) was transformed with derivatives of pSPB15 bearing the indicated *orc1* alleles and incubated at 30 °C. Serially diluted cells were spotted on the indicated selective media and further incubated at 30 °C. (**b**) Flow cytometry analysis. YHK26 (*orc1-161 his3::pRS403*), YHK27 (*orc1-161 his3::ORC1-HS*), YHK28 (*orc1-161 his3::orc1 K362A-HS*), and YHK29 (*orc1-161 his3::orc1 R367A-HS*) were grown at 23 °C to early log phase and then further incubated at 35 °C. Samples were taken at the indicated time points after the temperature shift. (**c**) ChAP assay. Cells of YHK26 (*orc1-161 his3::pRS403*), YHK33 (*orc1-161 his3::ORC1-His*_*12*_), YHK34 (*orc1-161 his3::orc1 K362A-His*_*12*_), and YHK35 (*orc1-161 orc1 R367A-His*_*12*_) were grown at 23 °C and further incubated at 35 °C. The amounts of indicated chromosomal loci crosslinked to wild-type or mutant Orc1-His_12_ were quantified and shown as relative enrichment compared with those using YHK26 (n = 3; mean ± SE).

To determine whether any of the residues in the EOS play a critical role *in vivo*, we performed a plasmid-shuffle assay using an *orc1* disruptant pre-complemented with a *URA3* plasmid bearing *ORC1* as a tester strain. If the *URA3* plasmid was swapped for a *LEU2* plasmid bearing *ORC1*, it still complemented the *orc1* disruption, but if it was swapped for a plasmid bearing *orc1 K485T*, an ATP-binding Walker A mutant, complementation was abolished (Fig. 3a), consistent with previous studies^30^,^31^. Notably, alanine-scanning experiments of the EOS using this assay revealed that *orc1 K362A* and *orc1 R367A* also failed to complement: these mutations resulted in slight residual activity and almost no activity, respectively (Fig. 3a). Both residues are highly conserved from yeast to human (Fig. 2b).

Given that ORC is essential for the cell cycle, we predicted that the *orc1 K362A* and *orc1 R367A* strains would be defective in cell-cycle progression. To test this, we performed a cell-cycle execution point analysis of the *orc1 K362A* and *orc1 R367A* mutants. Because *orc1 K362A* and *orc1 R367A* strains obtained by plasmid shuffling did not grow (Fig. 3a), we constructed conditionally lethal variants by integrating His–Strep II (HS)-tagged *orc1 K362A* or *orc1 R367A* into the *his3* locus of a temperature-sensitive *orc1-161* strain. Cells expressing only the *orc1-161* allele grow at the permissive temperature of 23 °C, but fail to grow at 30–37 °C due to a reduction in Orc1 protein level^32^,^33^,^34^. Addition of HS-tag sequences to endogenous *ORC1* did not significantly affect doubling time or cell-cycle progression (Supplementary Fig. S2). *orc1-161/ORC1-HS* cells grew normally at 35°C, but *orc1-161*/*orc1 K362A-HS* and *orc1-161*/*orc1 R367A-HS* cells both exhibited severe cell-cycle delays after shift to the non-permissive temperature (Fig. 3b). Cell cycle of most of those cells was arrested in G2/M phase at the non-permissive temperature, which is consistent with the fact that loss of *ORC1* function activates the DNA damage and spindle assembly checkpoint pathways^33^. Cell-cycle execution in *orc1-161/K362A-HS* cells was faster than that in *orc1-161/orc1 R367A-HS* cells. This is consistent with the results of the plasmid shuffle assay (Fig. 3a). ORC hexamers containing Orc1 K362A might have slight residual activity in initiation (see below). Hereafter, ORC hexamers containing Orc1 K362A or Orc1 R367A are referred to as ORC-1^K362A^ and ORC-1^R367A^, respectively,

Based on those results, a plausible idea is that ORC-1^K362A^ and ORC-1^R367A^ have less or no affinity for ARS. To test this possibility, we first established a chromatin affinity purification (ChAP) assay in which His_12_-tagged Orc1 (Orc1-His_12_) is crosslinked with DNA and is recovered using a pull-down method under a denatured condition. The previously published Orc1 ChIP assay under a native condition underestimates Orc1-ARS binding in G1 phase due to steric inhibition of immunoprecipitation by ORC-bound replication proteins^34^. Integration of the *ORC1*-*His*_*12*_ gene into the *his3* locus of the *orc1-161* strain suppressed temperature sensitive phenotype (Supplementary Fig. S3), indicating that Orc1-His_12_ is active *in vivo*. Using this strain, association of Orc1-His_12_ with *ARS1* and *ARS609*, a representative early and late origin, respectively, was observed in a His_12_-tag-dependent manner (Fig. 3c). In contrast, association of Orc1-His_12_ with *URA3*, a representative non-origin locus, was only minimal (Fig. 3c), as shown by Orc1 ChIP^34^.

Next, similar assays were performed using strains bearing mutant *orc1-His*_*12*_. Orc1 K362A-His_12_ and Orc1 R367A-His_12_ were impaired in binding to *ARS1* and *ARS609* although Orc1 K362A-His_12_ retained slight residual activity (Fig. 3c). These results are well consistent with the data shown above (Fig. 3a,b). Binding of these mutant Orc1-His_12_ to *URA3* was kept to only a minimum (Fig. 3c), suggesting that it is unlikely that DNA replication promiscuously initiates from non-ARS regions which mutant ORC binds less sequence-specifically although the possibility of firing at unidentified non-ARS regions is not completely denied. A likely idea is that replication process on ARS is impaired in these mutant cells. Taken together, we conclude that Orc1 Lys-362 and Arg-367 residues have a crucial role for ARS binding of Orc1 *in vivo.*

### Rapid purification of ORC using a mammalian overexpression system

**Figure 4:**
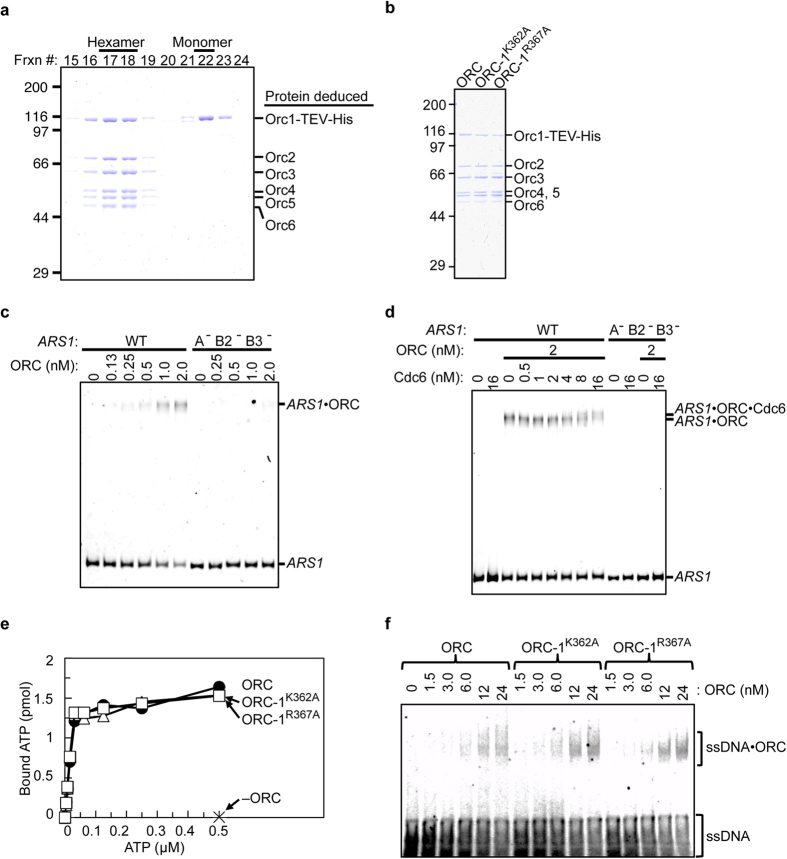
*In vitro* activities of ScORC purified from a mammalian overexpression system. (**a**) Gel-filtration analysis of affinity-purified ScOrc1-TEV-His co-overexpressed with ScOrc2/3/4/5/6 in 293T cells. Fractions were analyzed by 9% SDS-PAGE, followed by Coomassie staining. Gel positions of the ORC subunits are indicated. (**b**) Purified wild-type ORC, ORC-1^K362A^, and ORC-1^R367A^ (1.2 pmol each) were analyzed by 9% SDS-PAGE, followed by Coomassie staining. (**c**) EMSA of Cy5-labeled DNA with wild-type ORC. DNA fragments contained wild-type (WT) or mutant (A^−^ B2^−^ B3^−^) *ARS1*. Protein concentrations are indicated. (**d**) EMSA of Cy5-labeled DNA, as in panel **c**, in the presence of wild-type ORC and Cdc6. (**e**) Affinities for ATP. Wild-type ORC or mutants (2.4 pmol each) were incubated with the indicated amount of [α-^32^P]ATP, and bound nucleotide was quantified by filter binding assay. (**f**) EMSA of Cy5-labeled ssDNA with wild-type ORC or mutants.

Previously published protocols for purification of recombinant ORC for EM studies and quantitative biochemistry involve co-overexpression of ORC components^5^,^28^,^35^,^36^. For preparation of mutant ORC, these methods are time-consuming due to the necessity of combining baculoviruses expressing multiple ORC subunits and the number of traditional fractionation steps that must be performed without affinity tags. To accelerate mutational analyses of ORC *in vitro*, we developed a novel method for overexpression and purification of ORC in mammalian cells. In this system, ORC-overproducing plasmids, once mutagenized and sequenced, can be directly subjected to efficient co-transfection into mammalian cells^37^, thereby eliminating the need to construct and amplify baculoviruses. In addition, the use of an affinity column enabled omission of several purification steps. Using our new method, we obtained 0.72 mg of wild-type ORC from 400 ml of medium with a purity of ~90%, comparable to the yield from insect cells (Fig. 4a,b).

To determine whether this ORC preparation could be used for downstream applications (e.g., for biochemical assays), we analyzed the affinity of the purified ORC for *ARS1* DNA by electrophoretic mobility shift assay (EMSA) using a fluorescent *ARS1* fragment and non-labeled competitor DNA. The results revealed that our purified ORC retained sequence-specific affinity for *ARS1* (Fig. 4c). Furthermore, when Cdc6 was added to this assay, the ORC–Cdc6 interaction was detectable as a Cdc6-dependent supershift of the ORC–*ARS1* complex (Fig. 4d). These results are consistent with previous studies using ORC purified from baculovirus-transfected cells^38^, indicating that our novel ORC preparation is as useful for downstream applications as conventionally obtained material.

Next, we independently purified ORC-1^K362A^ and ORC-1^R367A^. The purity of the mutant ORCs was comparable to that of the wild-type ORC (Fig. 4b).

### Affinities of ORC-1^K362A^ and ORC-1^R367A^ for ATP and single-stranded (ss) DNA

To determine whether K362A and R367A mutations affect overall ORC activities, we first analyzed nucleotide binding by a nitrocellulose membrane-retention assay. The affinities of ORC-1^K362A^ and ORC-1^R367A^ for ATP were similar to that of wild-type ORC (Fig. 4e and Table 1).

ORC binds non-specific ssDNA *in vitro* in an ATP-independent manner^39^. We tested the mutant ORCs for this affinity by EMSA using 39-mer fluorescent ssDNA and competitor dsDNA. Wild-type ORC, ORC-1^K362A^, and ORC-1^R367A^ all bound to ssDNA in an ORC concentration-dependent manner (Fig. 4f). These results suggest that Orc1 Lys-362 and Arg-367 residues are not required for ssDNA binding.

### ORC-1^K362A^ and ORC-1^R367A^ are defective in ARS binding

**Figure 5:**
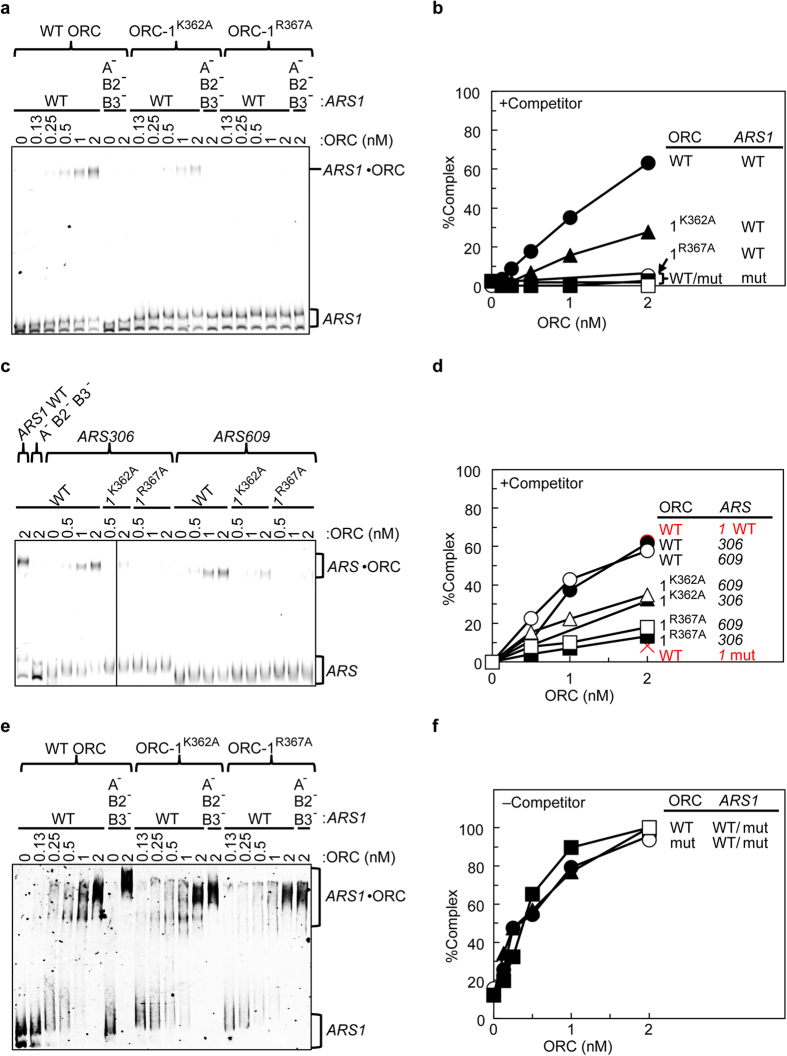
Affinities of ScORC for dsDNAs. (**a**) EMSA of Cy5-labeled wild-type (WT) or mutant (A^−^ B2^−^ B3^−^) *ARS1* DNA with wild-type or mutant ORC in the presence of competitor DNA. (**b**) Quantified results from panel (**a)** are shown. (**c**) EMSA of Cy5-labeled *ARS306* or *ARS609* DNA with wild-type or mutant ORC in the presence of competitor DNA. Wild-type (WT) or mutant (A^−^ B2^−^ B3^−^ or mut) *ARS1* was also used as controls. (**d**) Quantified results from panel **c** are shown. (**e**,**f**) EMSA of Cy5-labeled DNA in the absence of competitor DNA. DNAs were the same as in panels (**a**,**b**).

Next, we examined the ARS-binding activities of the mutant ORCs by EMSA using Cy5-labeled *ARS1* and unlabeled competitor DNA. Both ORC-1^K362A^ and ORC-1^R367A^ had a lower affinity for *ARS1* than wild-type ORC: binding of K362A was low but detectable, whereas the binding of R367A was almost undetectable (Fig. 5a,b). Similar results were obtained using *ARS306* and *ARS609*, which are representative early and late origins, respectively (Fig. 5c,d), suggesting that the dependency of ARS binding upon Orc1 Lys-362 and Arg-367 is general, rather than an *ARS1*-specific phenomenon. The relative residual activities of Orc1 K362A and Orc1 R367A were consistent with those observed *in vivo* (Fig. 3), suggesting that the *in vivo* phenotypes of these mutants can be essentially explained by the defects in ARS-binding activity observed *in vitro*.

Next, we examined the non-specific DNA binding activity of ORC. As noted above, omission of competitor DNA eliminates the sequence specificity of ORC-DNA binding^13^. When EMSA was performed in the absence of competitor DNA, ORC-1^K362A^ and ORC-1^R367A^ both retained significant affinity for mutant *ARS1* DNA (Fig. 5e,f). This result indicates that the binding mechanisms of ORC with non-specific and ARS DNA are mediated by at least partially independent mechanisms in terms of their requirement for the Orc1 Lys-362 and Arg-367 residues (see below).

### Role for Orc1^301–400^ in recognition of ARS

**Figure 6:**
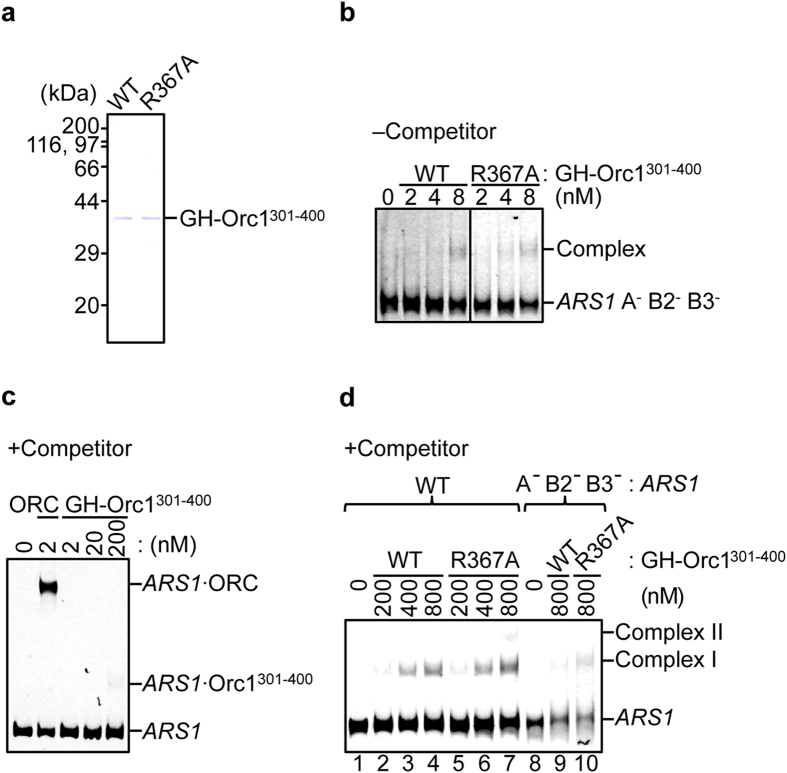
Affinities of Orc1^301–400^ for dsDNAs. (**a**) Wild-type and mutant GH-tagged Orc1^301–400^ (WT and R367A; 0.5 μg each) were analyzed by 12% SDS-PAGE, followed by Coomasie Brilliant Blue staining. (**b**) EMSA of Cy5-labeled mutant (A^−^ B2^−^ B3^−^) *ARS1* DNA with wild-type or mutant GH-Orc1^301–400^ in the absence of competitor DNA. (**c**) EMSA of Cy5-labeled wild-type *ARS1* DNA with ORC hexamer or GH-Orc1^301–400^ in the presence of competitor DNA. (**d**) EMSA of Cy5-labeled wild-type (WT) or mutant (A^−^ B2^−^ B3^−^) *ARS1* DNA with wild-type or mutant GH-Orc1^301–400^ in the presence of competitor DNA.

To investigate if Orc1 binds to ARS DNA via EOS *in vitro*, Orc1^301–400^ polypeptides with or without R367A substitution were purified as a GST-His (GH) tagged form up to near homogeneity (Fig. 6a). Orc1^301–400^ bears a part of the linker domain including EOS, but the AAA+ and WH domains are excluded (Fig. 1c). In the absence of competitor DNA, both wild-type and mutant GH-Orc1^301–400^ exhibited affinity for mutant *ARS1* DNA (Fig. 6b), indicating that Orc1^301–400^ basically has EOS-independent non-specific DNA binding activity. In the presence of competitor DNA, wild-type GH-Orc1^301–400^ retained affinity for wild-type *ARS1* DNA, but its affinity was at least 10^2^-fold lower than that of ORC hexamer (Fig. 6c). This is consistent with the fact that ORC subcomplexes lacking one of Orc1/2/3/4/5 subunits are severely impaired in binding to ARS^24^.

Intriguingly, competitor DNA effectively reduced binding of wild-type GH-Orc1^301–400^ to mutant *ARS1* and wild-type ARS sequence-dependent binding was evidently observed (Fig. 6d, lanes 4 and 9). These results suggest that Orc1^301–400^ contains *ARS1-*specific binding activity even though its affinity is low. When similar assay was performed using GH-Orc1^301–400^ bearing R367A, interaction with wild-type *ARS1* DNA was slightly enhanced compared with wild-type GH-Orc1^301–400^ and even a supershift was observed (Fig. 6d, lanes 2–4 vs. lanes 5–7). Competitor DNA did not inhibit binding of GH-Orc1^301–400^ R367A to mutant *ARS1* DNA as effectively as it did binding of wild-type GH-Orc1^301–400^ (Fig. 6d, lane 10). These results can be explained by a possibility that R367A mutation in GH-Orc1^301–400^ reduced sequence specificity of the polypeptide binding and stimulated abnormal binding modes of the polypeptide with DNA. In ORC hexamers, if Arg367-dependent recognition of *ARS1* is impaired, abnormal binding modes with DNA might be conformationally inhibited by other domains of Orc1 or another ORC subunit(s) (see Discussion). Taken together, these results suggest that Orc1^301–400^ solely recognizes ARS sequence with low affinity and that Arg-367 residue stimulates sequence specific binding mode of Orc1^301–400^.

## Discussion

In this study, we used a combination of *in silico* screening, yeast genetics, and biochemical analyses using mutant ORC proteins to identify Orc1 residues in the EOS that are essential for specific ORC hexamer–ARS binding. The EOS is located in a putative intrinsically disordered region rich in basic residues, between the BAH and the AAA+ domains of Orc1, which had not been previously proposed to be a DNA-binding domain. The critical residues identified in the EOS are highly conserved among eukaryotes, but not in the eubacterial and archaeal orthologs (Fig. 2; Supplementary Data S1; Supplementary Fig. S1). To our knowledge, this is the first evidence of a conserved structure–function relationship relevant to origin DNA binding by eukaryotic ORC. Intrinsically disordered basic regions in certain proteins facilitate or modulate selectivity by engaging in specific interactions with DNA^40^, which is consistent with our results (Figs 3c and 5). Taken together, we suggest that the EOS directly involves in effective and specific recruitment of ORCs onto ARSs. This mechanism might underlie the previous *in vivo* observation suggesting that the number of the total ORC molecules in a cell and the number of ORC-bound ARSs in a G1-phase cell are similar^41^,^42^: EOS might effectively support recruitment of ORC molecules to ARSs. Moreover, although the above model was constructed on the basis of findings in *S. cerevisiae*, it is consistent with a previous subcellular localization study in human cells showing that deletion of a region in HsOrc1 including both the nuclear localization signal and the entire EOS-homologous motif abolishes colocalization of HsOrc1 foci with DAPI^43^. We do not exclude a possibility that another region as well as EOS is important in regulation of specific localization of ORC.

**Figure 7:**
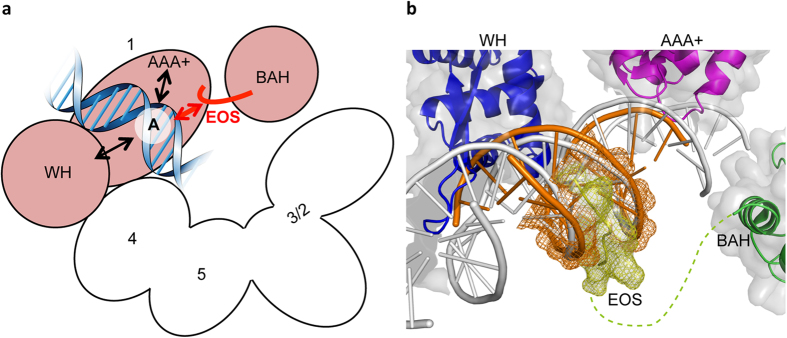
Model for EOS-mediated origin recognition in eukaryotic ORC. (**a**) A cartoon of ORC illustrating recognition of the A element via EOS and other domains in a mutually supportive manner. Orc1 and Orc2/3/4/5 are shown in pink and white, respectively. For clarity, Orc6 and the WH domains of Orc2/3/4/5 are omitted. (**b**) An atomic model predicts putative location of EOS. EOS (yellow) complexed with ACS DNA (orange) was computer-modeled and superimposed on the crystal structures of *S. solfataricus* Orc1-3 bound to DNA (PDB ID: 2QBY)^21^ and ScOrc1 BAH domain (green; PDB ID: 1M4Z)^8^. The AAA+ and WH domains and DNA in the crystal structure are shown in magenta, blue, and gray, respectively. See Discussion for details.

It is unlikely, however, that the EOS is sufficient for ORC hexamer to successfully bind an ARS, as affinity of Orc1^301–400^ for *ARS1* is at least 10^2^-fold weaker than that of ORC hexamer (Fig. 6c). We suggest that EOS-flanking region in Orc1^301–400^ and other domain(s), as well as EOS, play mutually supportive roles to achieve high-affinity ARS binding, as with the WH and AAA+ domains of archaeal ORC (but not eubacterial DnaA)^22^. This is consistent with our finding that ORC-1^K362A^, ORC-1^R367A^, and GH-Orc1^301–400^ R367A retain non-specific DNA binding in the absence of competitor DNA (Figs 5e,f and 6b). Indeed, the ARS region bound to ORC (i.e., from A to B1) is longer than the EOS. In the archaeal ORC ortholog Orc1-1, mutations in an origin-binding site of AAA+ domain impair origin sequence specificity but only slightly reduce affinity for origin DNA^22^, which is likely similar to our observation that GH-Orc1^301–400^ bearing R367A exhibited non-specific binding activity even in the presence of competitor DNA (Fig. 6d). Thus, we suggest a model in which EOS directly recognizes the A element of ARS (Fig. 7), although we do not exclude an alternative model in which EOS might regulate direct interaction between somewhere in Orc1^301–400^ and the A element.

Taken together, we suggest the following model for recognition of replication origins and initiation thereon. First, ORC scans DNA non-specifically in an EOS-independent manner. When ORC reaches an ARS, unstructured EOS recognizes the A element via weak but specific affinity, followed by stabilization of the binding by other structured domains such as WH and AAA+ (Fig. 7a). This stabilization assumes certain steric configuration by EOS-mediated specific DNA recognition. In this case, EOS is likely to contact DNA from the opposite side of the WH and AAA+ domains: a minor groove might be involved in this contact (Fig. 7b). The Orc1 Arg-367 residue makes a greater contribution to this process than Lys-362. This recognition might stimulate conformational changes in the overall ORC structure, particularly Orc1. In the ORC–ARS intermediate, the conformationally altered ORC transiently bends the DNA to pull in the B1 element and facilitate its recognition, presumably via Orc2, 3, or 5 (Fig. 1a). This step, which confirms that the bound DNA region lies within an ARS, may be enhanced by additional conformational changes that occur upon ORC–Cdc6 binding. The resultant ORC–Cdc6–ARS complex ensures ARS-specific recruitment of the Cdt1–MCM complex. This model is consistent with the observation that ORC-1^K362A^ and ORC-1^R367A^ retain non-specific DNA-binding activity (Fig. 5e,f), as well as the results of a recent single-molecule study showing that ORC can slide along non-specific DNA sequences to reach an ARS region^44^. Also, this model explains a possible mechanism underlying the observation that GH-Orc1^301–400^ bearing R367A binds DNA non-specifically, but ORC-1^R367A^ does not bind to ARS. The different degree of contribution by Lys-362 and Arg-367 residues in the model are consistent with the differences we observed in the residual activities of ORC-1^K362A^ and ORC-1^R367A^, both *in vitro* in the ARS binding assay (Fig. 5a–d) and *in vivo* in the plasmid-shuffle, temperature-shift/flow cytometry, and ChAP assays (Fig. 3). Furthermore, this model agrees with the results of previous *in vivo* comprehensive mutagenesis studies using *ARS1* and *ARS307, in vitro* footprinting using A^−^ and B1^−^ mutants, and our structural model^12^,^14^,^28^,^42^. Notably, our model explains why the ACS is more highly conserved throughout the genome than the B1 element, but still not sufficient for ORC binding^42^,^45^. Also, this model is consistent with previously reported models in which ORC bends the ARS^24^,^28^,^29^, analogous to bending of DNA by the intrinsically disordered C-terminus of histone H1^46^.

Origin sequences in eukaryotes are divergent compared with those in eubacteria and archaea^15^,^16^,^47^, but it remains unclear why the mechanisms of origin recognition by eukaryotic ORC could have diversified despite the fact that WH and AAA+ domains in ORC are highly conserved. Because EOS-homologous motifs contain slight deviations, except for the two essential residues in ScOrc1 (Fig. 2b), we hypothesize that eukaryotic ORC evolved its origin recognition strategy via mutation of the EOS and/or flanking disordered region. This is consistent with the reported robustness of disordered polypeptides with respect to mutation and the role of disordered regions in defining DNA sequence-specific contacts^48^,^49^. This hypothesis may provide a key to solving species-specific mechanisms of ORC in other organisms.

Another noteworthy finding of this study is that the EOS-homologous motif in Hs Orc1 is conserved even though a G-rich RNA/ssDNA-binding domain is present immediately upstream of the AAA+ domain, a region flanking the EOS-homologous motif (Fig. 2b)^50^. Replication origins in human cells associate with a G quadruplex (G4)-forming consensus motif 51; thus there is an intriguing coincidence between the location and function of the G-rich RNA/ssDNA-binding domain. The EOS-homologous motif and the G-rich RNA/ssDNA-binding domain in HsOrc1 may have distinct, specific roles in recognition of origin DNA.

A similar idea could be extended to the possible roles of the EOS-homologous motifs in *S. pombe, Drosophila*, and human, in which the unique N-terminal extension of SpOrc4 and the transcription factor-homologous domain in DmOrc6 and HsOrc6, respectively, are solely responsible for DNA binding (Fig. 2b)^17^,^23^,^27^,^52^. We suggest that the EOS-homologous motifs of Orc1 in these species might play a distinct role in DNA binding and formation of active nucleoprotein complexes even when SpOrc4, DmOrc6, and HsOrc6 are bound to origin DNA. Consistent with this idea, SpORC binds to DNA in at least two steps *in vitro*, of which only the first step involves the extension of the SpOrc4 subunit^53^. In *S. cerevisiae*, Orc6 is dispensable for ORC–ARS binding and contains a long insertion not found in other eukaryotes^7^,^24^.

It has been reported that the BAH domain in metazoan Orc1 regulates ORC chromatin association, cell cycle progression, and development via interaction with a histone H4K20me2^54^. However, core histone-mediated regulation of ORC-DNA binding may be diversified among species. For example, ScOrc1 and SpOrc1 do not bind to H4K20me2^54^. In *S. cerevisiae*, stabilization of ORC-ARS binding by nucleosomes *in vitro* is independent of the BAH domain of Orc1^10^ even though the BAH domain in ScOrc1 stabilizes *in vivo* association of ORC to a subset of ARSs^9^. As this apparent complexity is of future interest, further dissection of ORC functional structures will pave the way to understand detailed mechanisms in ORC-nucleosome interactions.

During preparation of this manuscript, the crystal structure of a DNA-free, truncated form of DmORC was reported^55^. The reported structure is fully consistent with our model. The overall structures of ScORC and DmORC share a similar architecture^28^ and DmOrc1 also bears an EOS-homologous motif (Fig. 2b), which was eliminated in order to achieve crystallization.

## Methods

### Yeast techniques

Yeast media and cell synchronization were as described^56^. For plasmid-shuffle assay, medium was supplemented with 1 g/L 5-fluoroorotic acid (FOA). For flow cytometry, cells were stained with SYTOX Green (Life Technologies) and analyzed on a FACSCalibur using the CellQuest Pro software (BD Biosciences) as described previously^56^.

### Plasmids and yeast strains

Yeast pRS vectors and pSPB15 (*ORC1 ARS CEN LEU2*) were gifts from Dr. Bruce Stillman. The mammalian expression vector version 3–5^37^ was a gift from Dr. Hisao Masai. Site-directed mutagenesis was performed using the QuikChange site-directed mutagenesis kit (Agilent) and verified by sequencing. pHK142 (*GH-ORC1*^*901–1200*^) and pHK143 (*GH-orc1*^*901–1200*^ *R367A*) were constructed by Gibson assembly using pGEX-6P-1 (GE Healthcare), followed by site-directed mutagenesis. Yeast strains are listed in Supplementary Table S1.

### Computational analysis

The secondary structure of ScOrc1 was predicted using PROFphd^57^. Multiple alignment of ScOrc1^201–420^ was generated using COBALT^58^ with a semi-automated pipeline: the raw data of a BLAST search were used as the initial data set, and the duplicated or truncated sequences in the alignment were removed manually. Structure of ScOrc1^362–367^ complexed with an 11-bp ACS DNA was modeled using MacroModel (Schrödinger). Graphical representations of the 3D model and the flow cytometry data were generated in PyMOL and R/Bioconductor using the *ggplot2* package, respectively.

### Chromatin affinity precipitation (ChAP)

ChAP experiments were carried out as described^34^,^56^,^59^ with some modifications. Briefly, 25-ml culture of cells was incubated at 23 °C until A_600_ reached 0.5, followed by further incubation at 35 °C for 2 h. Cells were crosslinked, spheroplasted, and lyzed. Chromatin-containing insoluble materials were pelleted, re-suspended, and sonicated to yield a mean DNA size of 500 bp. A cleared lysate was prepared, mixed with 2.5 volumes of buffer B (20 mM Tris-HCl [pH 7.5], 1% Triton X-100, 8 M urea, 500 mM NaCl, 2 mM PMSF, and 10 mM imidazole), and subjected to a pull down method using Dynabeads His-tag (Invitrogen). The eluate with buffer E (50 mM Tris-HCl [pH 7.5], 500 mM imidazole, 1% SDS, 10 mM EDTA, 10 mM DTT, and 500 mM NaCl) was treated with 20 μg RNase A for 15 min at 37 °C and decrosslinked by incubation at 65 °C for 6 h. DNA was column-purified, subjected to qPCR, and quantified using the second derivative maximum method: the primers used were HK297 (GCTCCCTAGCTACTGGAGAATA) and HK298 (CTCTTCCACCCATGTCTCTTTG) for *URA3*, HK299 (TGTTTGTGCACTTGCCT) and HK300 (CATTGCGGTGAAATGGTAAA) for *ARS1*, and HK313 (TTTGTGAATTTAGCCAATTCCG) and HK314 (GCCATTGAATTATGCCGAGAG) for *ARS609*, respectively. As a control, 4% of the input sample was similarly processed.

### Proteins

ScCdc6 was overexpressed in *E. coli* and purified as described^5^,^28^,^38^. Overexpression and purification of ScORC will be described in detail elsewhere. Briefly, derivatives of the version 3–5 vector^37^ carrying *ORC1-TEV-His, ORC2, ORC3, ORC4, ORC5*, and *ORC6* were transiently co-transfected to 293T cells by the PEI method^37^ ORC hexamer was affinity-purified and further fractionated using Superdex 200. GH-Orc1^301–400^ was overexpressed in *E. coli* BL21(DE3) bearing pCodonPlus (Agilent) and pHK142 or pHK143, and a cleared lysate was prepared. GH-Orc1^301–400^ was isolated by a pull-down method using Ni-Sepharose, and eluted by step gradient of imidazole. Composition of buffers was the same as that used for purification of ORC hexamer.

### ATP-binding assay

ATP bound to ORC was determined as described^31^,^60^ with some modifications. Briefly, ORC (2.4 pmol) and [α-^32^P]ATP were incubated for 5 min at 30 °C in 50 μl of buffer K (45 mM HEPES–KOH [pH 7.6], 4.5 mM magnesium acetate, 140 mM KCl, 9% [v/v] glycerol). The reaction mixture was filtered through a nitrocellulose membrane (Merck Millipore) pre-equilibrated with buffer K. The membrane was washed with buffer K and dried. Radioactivity retained on the membrane was quantified using a liquid scintillation counter.

### Electrophoretic mobility shift assay (EMSA)

EMSA with ds or ssDNA was carried out as described^38^,^39^ with minor modifications. A 290 bp segment containing wild-type *ARS1* or a mutant (A^−^ B2^−^ B3^−^) were PCR amplified as described^38^. Fragments carrying *ARS306* or *ARS609* were similarly amplified using primers HK278 (CCCACATGTAAGCTTAACTTCTTCGTGAGGAAGGAAAGTG) and HK279 (CCGACGTCAGATCTTAATTTATCTCATGAAGTAATGATAC) for *ARS306*, and HK280 (CCCACATGTCAATTTAGTATATTACTGTATATCTAGTTC) and HK281 (CCGACGTCGTTAAAAACAGAAAAGTAAAAATTCCGATCTTG) for *ARS609*. For ssDNA binding, DL11 (GTTACCATGGCATCGAGTTCTTCAACAAGACTACAATGG)^39^ was used. These DNAs were labeled with Cy5-ddUTP using terminal transferase. ORC and Cdc6 were incubated with labeled DNA (1.6 nM as a fragment for Cy5-ARS; 3 nM for Cy5-DL11) and 300 ng of GC-rich competitor^38^ for 10 min at room temperature in 5 μl of binding buffer (25 mM HEPES–KOH [pH 7.6], 100 mM potassium glutamate, 5 mM magnesium acetate, 5 mM calcium chloride, 5 mM DTT, 5% [v/v] glycerol, 0.1% [v/v] Triton X-100, 2 mg/ml BSA, and 1 mM ATP). DNA was separated by 4% 29:1 polyacrylamide gel electrophoresis, and fluorescent signals were detected in an ImageQuant LAS4010 imager (GE Healthcare).

## Additional Information

**How to cite this article**: Kawakami, H. *et al.* Specific binding of eukaryotic ORC to DNA replication origins depends on highly conserved basic residues. *Sci. Rep.* **5**, 14929; doi: 10.1038/srep14929 (2015).

## Supplementary Material

Supplementary Information

Supplementary Data S1

## Figures and Tables

**Table 1:** Affinities of wild-type and mutant ORC for ATP.

	K_d_ (nM)	Stoichiometry
ORC	31	0.78
ORC-1^K362A^	35	0.82
ORC-1^R367A^	32	0.81

K_d_ and stoichiometry were estimated from Fig. 4e using Scatchard plots.
