# Supplementary material for: Specific binding of eukaryotic ORC to DNA replication origins depends on highly conserved basic residues
Source: Sci Rep. 2015 Oct 12;5:14929. doi: 10.1038/srep14929 (PMC4601075; doi:10.1038/srep14929)
Supplement: Supplementary Information [file srep14929-s1.pdf]

Supplementary Information for: Specific binding of eukaryotic ORC to DNA replication origins depends on highly conserved basic residues

Hironori Kawakami, Eiji Ohashi, Shota Kanamoto, Toshiki Tsurimoto, & Tsutomu Katayama

Supplementary Data legend

Supplementary Data S1. Full details of the multiple alignment shown in Fig. 2a. NCBI accession numbers are indicated in parenthesis.

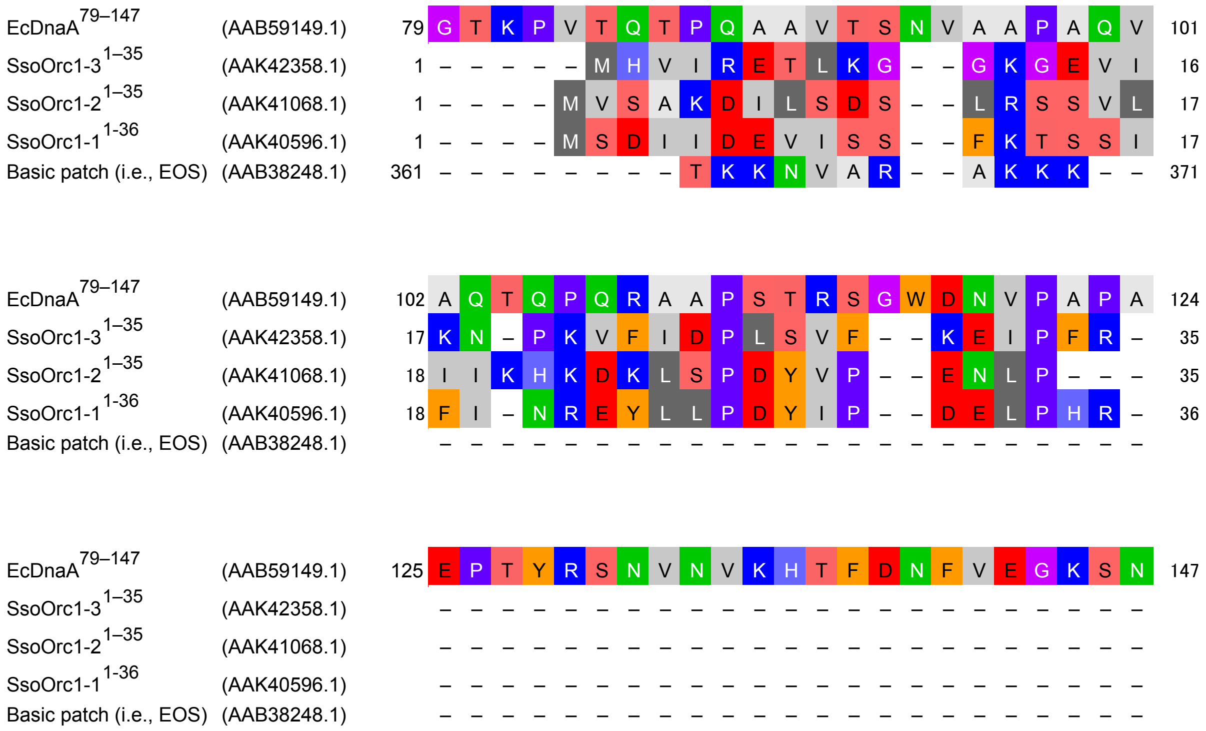

Supplementary Figure S1. Misalignment of the basic patch to ORC orthologs in eubacteria and archaea. The linker domain and flanking regions of DnaA in *E. coli* and upstream of AAA+ domains in Orc1-3, Orc1-2, and Orc1-1 (also known as Cdc6-3, Cdc6-2, and Cdc6-1, respectively) in *S. solfataricus* were compared with the basic patch (EOS). NCBI accession numbers are indicated.

a

Strain	Relevant genotype	Doubling time (min)
W303-1A	*ORC1*	101
YSK01	*ORC1-HS*	98

b

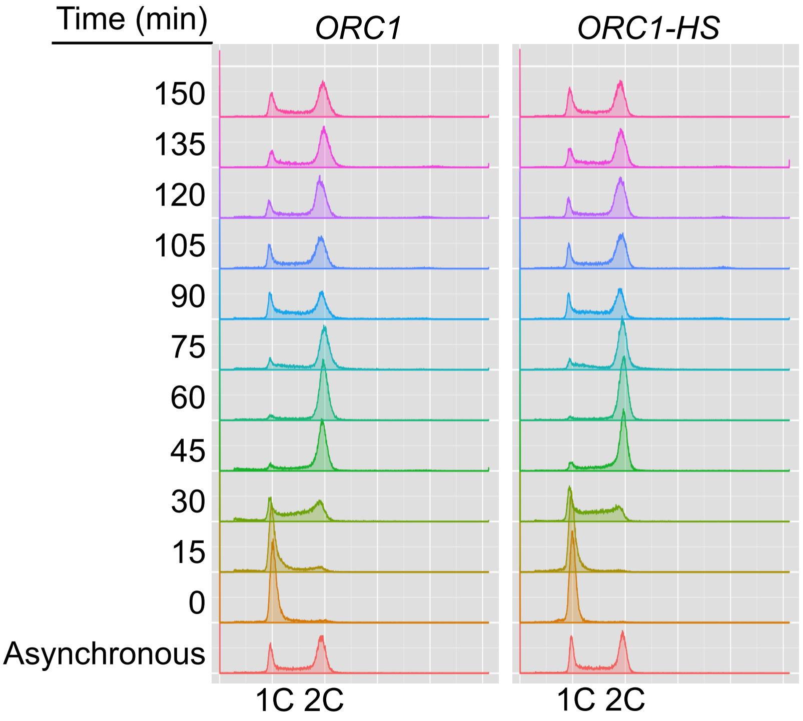

Supplementary Figure S2. No significant effect of HS-tag on Orc1 function. (**a**) Doubling time at 30°C in YPD medium. (**b**) Strains W303-1A (*ORC1* wild type) and YSK01 (*ORC1-HS*) were grown at 30°C in YPD medium up to mid-log phase, synchronized in G1 phase by incubation with α-factor, and released at the same temperature. Cell-cycle progression was monitored by flow cytometry.

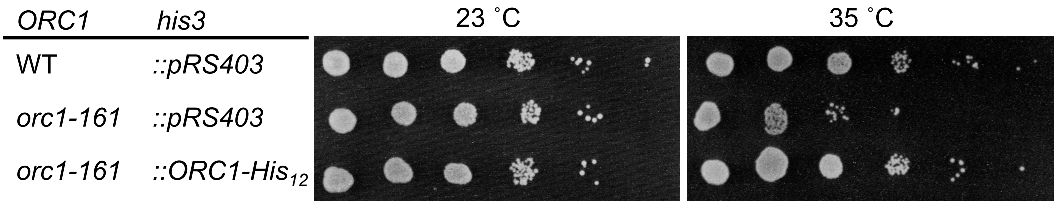

Supplementary Figure S3. His$_{12}$-tagged Orc1 is active *in vivo*. Strains YHK20 (*ORC1 his3::pRS403*), YHK26 (*orc1-161 his3::pRS403*), and YHK27 (*orc1-161 his3::ORC1-His$_{12}$*) were serially diluted, spotted, and incubated at indicated temperatures.

Supplementary Table S1. Yeast strains used in this study.

Name	Relevant genotype	Reference or source
W303-1A	*MAT***a** *leu2-3,112 his3-11,15 trp1-1 can1-100 ade2-1 ura3-1*	[1]
YB838	W303-1A *orc1::hisG* pSPB16 (*ORC1 ARS CEN URA3*)	Bruce Stillman
OAy422	*orc1-161 bar1* (W303-1A background)	Stephen P. Bell[2]
YSK01	W303-1A *ORC1-HS::His3MX6*	This study
YHK26	OAy422 *his3::pRS403*	This study
YHK27	OAy422 *his3::ORC1-HS (HIS3)*	This study
YHK28	OAy422 *his3::orc1 K362A-HS (HIS3)*	This study
YHK29	OAy422 *his3::orc1 R367A-HS (HIS3)*	This study
YHK33	OAy422 *his3::ORC1-*His_{12} *(HIS3)*	This study
YHK34	OAy422 *his3::orc1 K362A-*His_{12} *(HIS3)*	This study
YHK35	OAy422 *his3::orc1 R367A-*His_{12} *(HIS3)*	This study
SH5589	W303-1A *bar1::hisG*	Hiroyuki Araki via Satoshi Harashima and NBRP
YHK20	SH5589 *his3::pRS403*	This study

Supplementary References

1. Thomas, B.J. & Rothstein, R. Elevated recombination rates in transcriptionally active DNA. *Cell* **56**, 619-30 (1989).
2. Aparicio, O.M., Weinstein, D.M. & Bell, S.P. Components and dynamics of DNA replication complexes in S. cerevisiae: redistribution of MCM proteins and Cdc45p during S phase. *Cell* **91**, 59-69 (1997).
